# Tools for Supporting the MCH Workforce in Addressing Complex Challenges: A Scoping Review of System Dynamics Modeling in Maternal and Child Health

**DOI:** 10.1007/s10995-022-03376-8

**Published:** 2022-02-21

**Authors:** Isabella Guynn, Jessica Simon, Seri Anderson, Stacey L. Klaman, Amy Mullenix, Dorothy Cilenti, Kristen Hassmiller Lich

**Affiliations:** 1 Department of Maternal and Child Health, National MCH Workforce Development Center, University of North Carolina at Chapel Hill, 412 Rosenau Hall, Chapel Hill, NC 27599 USA; 2 Department of Health Policy and Management, Gillings School of Global Public Health, University of North Carolina at Chapel Hill, 1105E McGavaran-Greenberg Hall, Chapel Hill, NC 27599 USA; 3 Health Systems Transformation, Association of Maternal and Child Health Programs, 1825 K Street NW, Suite 250, Washington D.C, 20006 USA; 4 Present Address: RTI Health Solutions, Research Triangle Park, NC 27709-2194 USA; 5 Family Health Centers of San Diego, 823 Gateway Center Way, San Diego, CA 92102 USA; 6 Department of Maternal and Child Health, Gillings School of Global Public Health, University of North Carolina at Chapel Hill, 402A Rosenau Hall, Chapel Hill, NC 27599 USA

**Keywords:** System dynamics, Causal loop, Scoping review, Simulation, Strategic planning

## Abstract

**Objectives:**

System Dynamics (SD) is a promising decision support modeling approach for growing shared understanding of complex maternal and child health (MCH) trends. We sought to inventory published applications of SD to MCH topics and introduce the MCH workforce to these approaches through examples to support further iteration and use.

**Methods:**

We conducted a systematic search (1958–2018) for applications of SD to MCH topics and characterized identified articles, following PRISMA guidelines. Pairs of experts abstracted information on SD approach and MCH relevance.

**Results:**

We identified 101 articles describing applications of SD to MCH topics. *Approach:* 27 articles present qualitative diagrams, 10 introduce concept models that begin to quantify dynamics, and 67 present more fully tested/analyzed models. *Purpose:* The most common purposes described were to increase understanding (n = 55) and support strategic planning (n = 26). While the majority of studies (n = 53) did not involve stakeholders, 40 included what we considered to be a high level of stakeholder engagement – a strength of SD for MCH. *Topics:* The two Healthy People 2020 topics addressed most frequently were early and middle childhood (n = 30) and access to health services (n = 26). The most commonly addressed SDG goals were “End disease epidemics” (n = 26) and “End preventable deaths” (n = 26).

**Conclusions for Practice:**

While several excellent examples of the application of SD in MCH were found, SD is still underutilized in MCH. Because SD is particularly well-suited to studying and addressing complex challenges with stakeholders, its expanded use by the MCH workforce could inform an understanding of contemporary MCH challenges.

**Supplementary Information:**

The online version contains supplementary material available at 10.1007/s10995-022-03376-8.

## Significance

*What is already known on this subject?* The MCH workforce faces challenges that are dynamic and complex. Existing workforce approaches commonly take narrow perspectives rather than acknowledging broad dynamics of larger systems.

*What this study adds?* This is the first known attempt to identify all published research using SD to study MCH topics. The MCH workforce will be able to use this scoping review to (a) learn about the value of SD approaches for their work, (b) identify examples of strong SD approaches in MCH, and (c) consider potential applications of SD approaches in their own practice or research.

## Objectives

Maternal and child health (MCH) is a far-ranging field encompassing issues of preterm birth, childhood obesity, sexually transmitted infections, and maternal mortality, among others. A common thread across MCH issues is the fact that they are both “complex and dynamic”, meaning they are caused by a system of interconnected factors (e.g., crossing socio-ecological levels) that continue to change over time (Kroelinger et al., 2014; Meadows & Wright, 2008). The contemporary MCH workforce faces tremendous challenges in responding to such persistent issues, particularly around making best use of limited resources and addressing issues of equity (Fanta, Ladzekpo et al. 2021; Mehta et al., 2021). This complexity requires MCH to work across system boundaries (e.g. organizational, disciplinary, geographic, life-course). Existing MCH strategies and approaches may be enhanced if the workforce embraced a systems perspective and integrated systems thinking tools into current practice (Kroelinger et al., 2014).

**Fig. 1:**
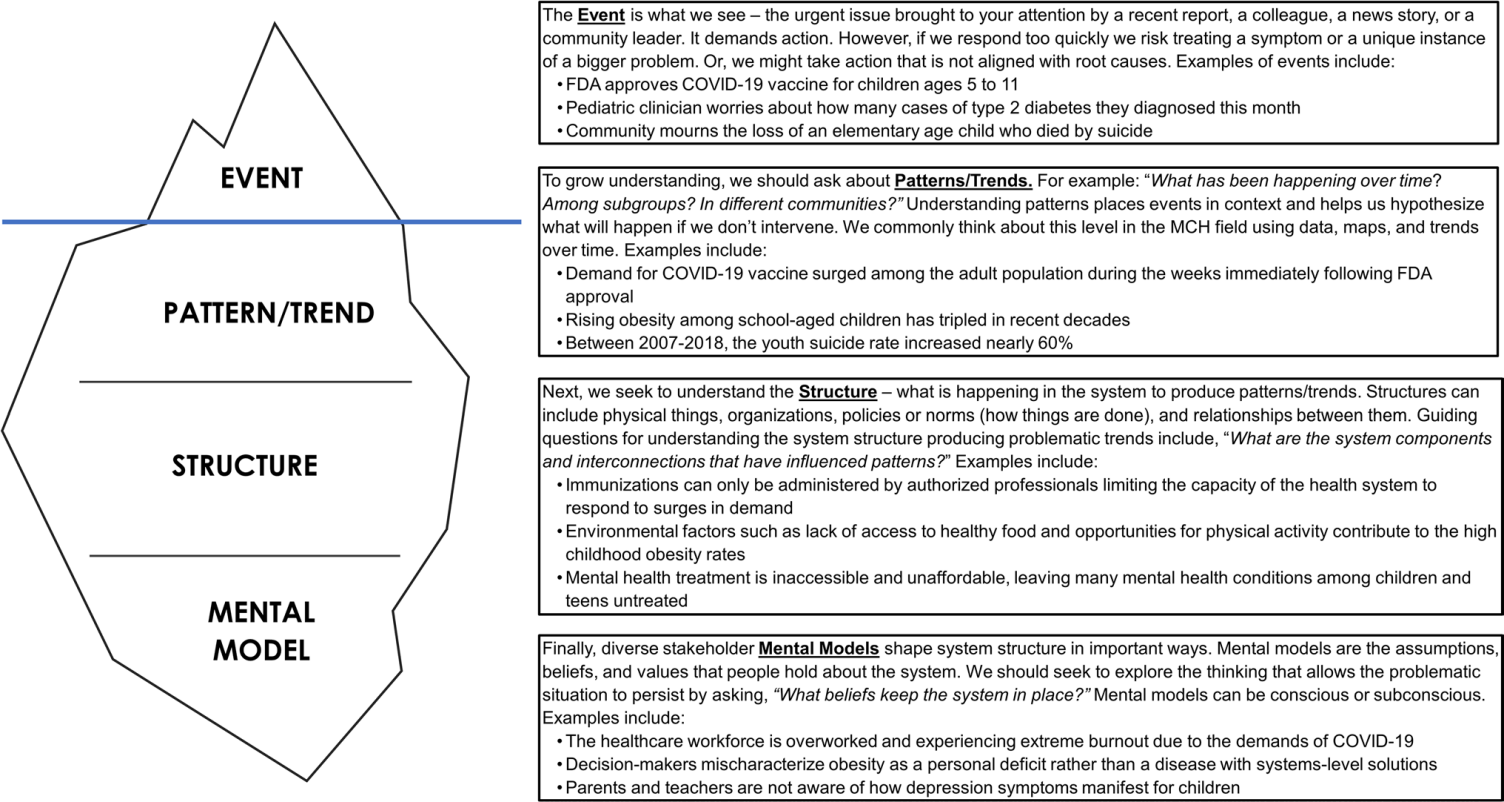
The Iceberg Model to System Thinking. The Iceberg Model is a common framework used to guide system thinking (Maani & Cavana, 2007). The top level (*“Event”*) represents the visible part of a problem, whereas the lower levels (*“Pattern/Trend”, “Structure”, and “Mental Model”*) consider more deeply elements of the system that produce the problem and leverage points for change

System Dynamics (SD) offers a set of tools and approaches for understanding behaviors of dynamic systems surrounding complex problems (Forrester 1961–1969, Meadows, 1999; Sterman, 2000, 2006; Homer & Hirsch, 2006; Meadows & Wright, 2008). SD acknowledges that problematic events or trends are produced because of the underlying system (Fig. 1); effectively addressing the problematic outcome requires an understanding of the system’s structure and the “mental models” of stakeholders keeping the problematic system in place (Maani & Cavana, 2007). Whereas many typical problem-solving approaches assume a consistent, linear relationship between variables, SD tools capture more realistic non-linearities caused by endogeneity (feedback) in causal relationships as systems respond to changes over time and delays in information and production (Haraldsson, 2004; Sterman, 1989). SD requires a more holistic understanding of key determinants of change over time, which aligns with the demands of the MCH environment.

**Fig. 2:**
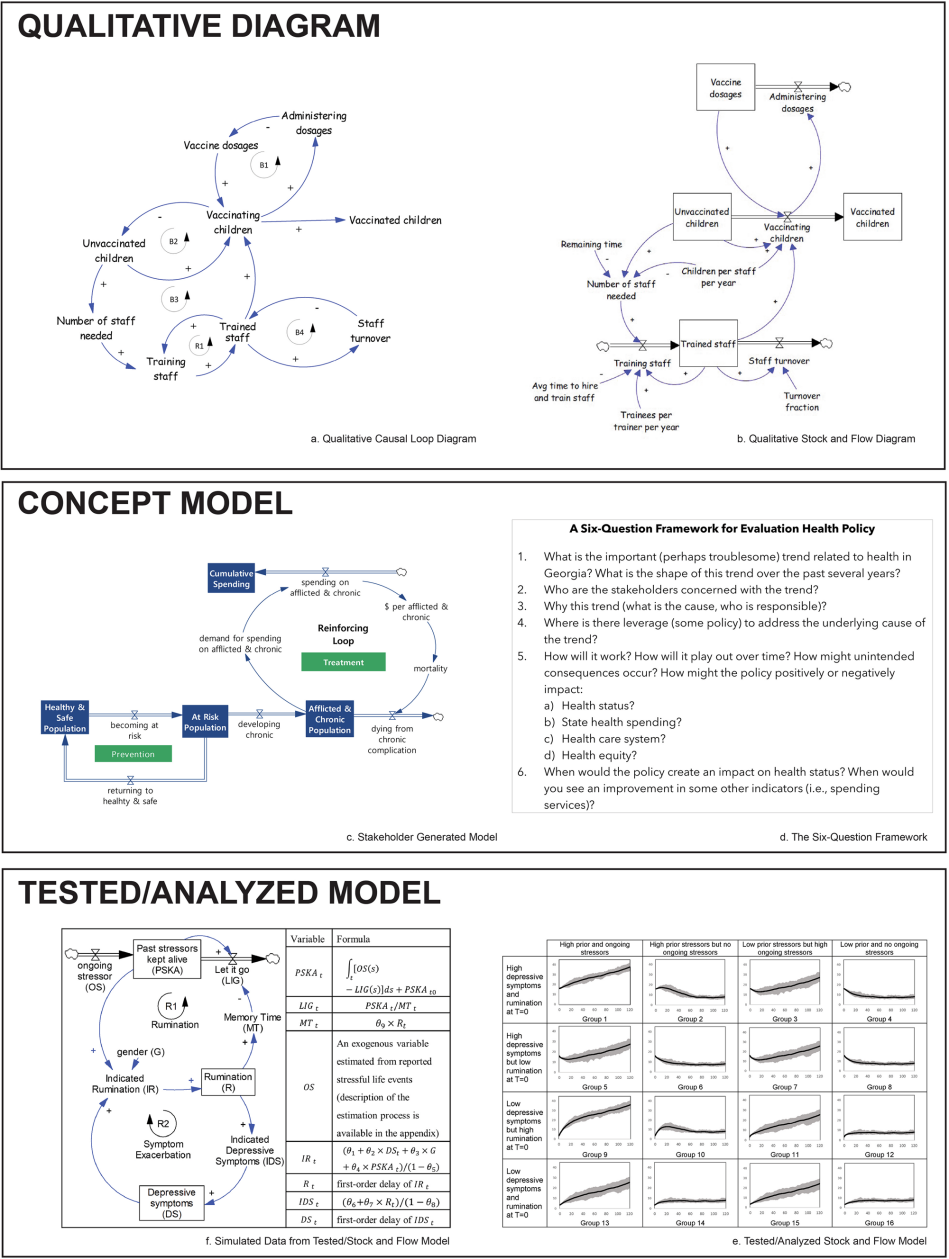
Figure 2 provides an example of each of the three main approaches seen in SD: Qualitative Diagrams, Concept Models, and Tested/Analyzed Models. The qualitative causal loop diagram (**a**) and a qualitative stock and flow diagram (**b**) were used to facilitate conversations among a group of stakeholders in Munar et al. (2015) regarding the impact that limited staff and clinic capacity has on getting children vaccinated. The concept model (**c**) is adapted from a model published in Minyard et al. (2014) that was used as a teaching tool with state-level policy makers. The concept model (**c**) along with the six-questions framework (**d**) were used as part of an iterative process in which policy makers interacted with SD models to “test” the impact of various policy scenarios through simulation modeling. Finally, in Hosseinichimeh et al. (2018), a tested/analyzed model (**e**) was built to understand and quantify the interactions between depressive symptoms, rumination, and stress in adolescent populations. The model was parameterized (see formulas in Fig. 2e) and primary-collected longitudinal data was inputted to estimate (via simulation modeling) the impact of prior stressors on current levels of depression for 16 different groupings of adolescents (**f**). *Note The building blocks of all SD models include **variables**, **arrows**, **polarity, feedback loops, stocks,** and **flows**. **Variables** are written as nouns or noun phrases with clear meaning when they increase or decrease over time. Thin **arrows** drawn between variables indicate that a change in the first variable triggers a change in the second variable over time, all else equal. The **polarity** of causal links is labeled “S” or “ + ” to indicate the variables change in the same direction (e.g., if the value of the first goes up, the value of the second also goes up); they are labeled “O” or “ − “ to indicate that the variables change in opposite directions (e.g., if the value of the first goes up, the value of the second goes down). **Feedback loops** are closed chains of causal linkages that reinforce (i.e., exacerbate) or balance (i.e., stabilize) changes over time. **Stocks** depict accumulations of focal variables whose value or level is determined by the balance of inflows and outflows over time; **flows** are notated using a double arrow with an hourglass and represent rates of change in and out of a stock over time. For more information on SD models, including how to “read” a model, see “Introduction to Systems Thinking” (Kim, 1999)

SD offers qualitative (e.g., causal loop diagrams) and quantitative (e.g., simulation models) tools. The application of SD tools can be organized into three approaches: qualitative diagrams, concept models, and tested/analyzed models; these approaches can be used independently or together as part of an iterative process, with qualitative diagrams being the starting point and tested/analyzed models being the end deliverable (Fig. 2). *Qualitative diagrams* are developed, often with stakeholders, to better understand complex, problematic trends that need to change. They facilitate conversations among diverse stakeholders by providing a tangible language for understanding structures and mental models surrounding persistent challenges. *Concept models* build upon qualitative diagrams by introducing preliminary (often hypothetical) numbers as model parameters and inputs. The model is then used to test hypotheses and explore impact of system feedback on outcomes of interest. The final iteration of SD approaches is *tested/analyzed models*. Model parameters are calibrated, often using historical data, until users feel confident in the model’s validity; from there, future trends and evidence can be generated using simulation modeling to test hypotheses, inform decision-making, and holistically study complex challenges.

SD is well-suited for practical application by the MCH workforce due to the feasibility of stakeholder engagement in the modeling process (Cilenti 2019). SD approaches encourage ‘group model building’, in which diverse stakeholders come together to create a shared map of the system that maintains a persistent problem (Vennix, 1996, 1999). SD tools and approaches facilitate productive dialogues across diverse stakeholders about causes of persistent MCH issues and possible system responses to different courses of action (i.e. practice/policy changes). For more information on SD, see Sterman (2000).

In the midst of widespread health systems transformation and movement toward equity-focused approaches in public health, MCH professionals have been embracing leadership roles in cross-sector collaborations. The time is ripe for SD to become more common in MCH practice and research as a way for the workforce to fully understand the systems in which they operate, predict unintended consequences of program and policy choices, and lead – informed by tools that enhance understanding of complexity as the field moves towards centering equity practice (Kroelinger et al., 2014). As such, this manuscript presents a scoping review of existing SD literature with application to MCH (Munn, 2018). We organize our findings into three domains: (1) SD approaches applied to MCH research, (2) purposes for which SD was used by MCH practitioners, and (3) MCH topics studied using SD.

## Methods

We attempted to identify all existing published research that used SD tools to study MCH topics between January 1958 and July 2018. Publications that met inclusion criteria were identified over four steps in the scoping review. These steps, discussed in detail in Online Appendix 1, were as follows: *Step 1* used three different search strategies in the Web of Science Core Citation Indexes (WOS) and PubMed to identify research using SD between January 1958 and July 2018. Works that did not have a health sciences or health services focus were conservatively filtered out based on title-screen in *Step 2.* In *Step 3*, pairs of authors reviewed titles and abstracts (if available) of all works meeting our SD and health criteria to identify those that demonstrated an application of SD methods. Finally, in *Step 4*, pairs of authors reviewed abstracts and full texts to identify works that were relevant to MCH and thus eligible to be included in this review. This review builds on a previously completed systematic search for SD applications in health. *Step 1* through *Step 3* reflect efforts accomplished as steps in this earlier search and *Step 4* reflects efforts specific to this scoping review; two members of the research team (KHL and IG) were among those who participated in the previous search process.

Information from the SD tools and approaches abstraction was double coded by a team of four authors who are experts in SD (KHL, IG, JS, SA). Information abstracted included: SD approach, model purpose, and level of stakeholder engagement*.* Three authors (DC, AM, SK) specializing in MCH practice, and collectively bring over 80 years of experience, conducted the MCH content abstraction. Information abstracted included: MCH-relevant Healthy People 2020 objectives addressed, MCH-relevant UN Sustainable Development Goals (SDGs) addressed, domestic- or global-focus, and utility for MCH research/policy/practice. All abstracted information was double coded and any discrepancies were resolved with the full abstraction team.

The abstracted information was chosen to be both practically useful to readers interested in seeking out works applying SD to MCH topics, and to gauge the extent to which SD/MCH researchers are studying high-priority MCH research topics. Definitions for SD approach, model purpose, level of stakeholder engagement, and utility for MCH research/policy/practice are provided in Table 1 footnotes. The authors chose the Healthy People 2020 topics to reflect current priority topics in US-domestic MCH research. The UN SDGs were chosen to reflect current international priority topics in MCH research. Two authors identified MCH-relevant Healthy People 2020 topics and SDGs using the project’s definition of MCH (see Online Appendix 2). The group then discussed and approved the included goals. Each work could be coded as studying any number of these goals, including none at all.

**Table 1:** Characteristics of maternal and child health studies using a system dynamics approach

Citation	Title	SD Approach	Model Purpose	Level of Stakeholder Engagement	CDC Healthy People 2020 Goals	UN Sustainable Development Goals (SDGs)	Domestic or Global Focus?	Utility for MCH Policy/ Practice?
(Abidin, Mamat et al. 2014)	Combating obesity through healthy eating behavior: a call for system dynamics optimization	Tested/ Analyzed Model	Informing policy	None	Adolescent health, early and middle childhood, nutrition and weight status		Global	**High**
(Ahmad, 2005a, 2005b)	The cost-effectiveness of raising the legal smoking age in California	Tested/ Analyzed Model	Informing policy	None	Adolescent health, health related quality of life and wellbeing, tobacco use, substance abuse	Strengthen prevention and treatment of substance abuse (3.5)	Domestic	**High**
(Ahmad, 2005a, 2005b)	Closing the youth access gap: The projected health benefits and cost savings of a national policy to raise the legal smoking age to 21 in the United States	Tested/ Analyzed Model	Informing policy	None	Adolescent health, health related quality of life and wellbeing, tobacco use, substance abuse	Strengthen prevention and treatment of substance abuse (3.5)	Domestic	**High**
(Ahmad & Billimek, 2007)	Limiting youth access to tobacco: Comparing the long-term health impacts of increasing cigarette excise taxes and raising the legal smoking age to 21 in the United States	Tested/ Analyzed Model	Informing policy	None	Adolescent health, health related quality of life and wellbeing, tobacco use, substance abuse	Strengthen prevention and treatment of substance abuse (3.5)	Domestic	**High**
(Anderson & Anderson, 1998)	HIV screening and treatment of pregnant women and their newborns: A simulation-based analysis	Tested/ Analyzed Model	Strategic planning (compare one plan to another)	None	HIV, sexually transmitted diseases, maternal, infant, and child health	End preventable deaths (3.2), End disease epidemics (3.3)	Domestic	Medium
(Barber & Lopez-Valcarcel, 2010)	Forecasting the need for medical specialists in Spain: Application of a system dynamics model	Tested/ Analyzed Model	Strategic planning (compare one plan to another)	**High**	Access to health services		Global	Low
(Batchelder et al., 2015)	A social ecological model of syndemic risk affecting women with and at-Risk for HIV in impoverished urban communities	Tested/ Analyzed Model	Increase understanding	**High**	HIV, injury and violence prevention, Mental and mental disorders, sexually transmitted diseases, social determinants of health	End disease epidemics (3.3)	Domestic	Low
(Batchelder & Lounsbury, 2016)	Simulating syndemic risk: Using system dynamics modeling to understand psycho-social challenges facing women living with and at-risk for HIV	Tested/ Analyzed Model	Increase understanding	**High**	HIV, injury and violence prevention, social determinants of health, health related quality of live and wellbeing, STDs, substance abuse	End preventable deaths (3.2), End disease epidemics (3.3), Strengthen prevention and treatment of substance abuse (3.5), Eliminate violence against women (5.2), Eliminate harmful practices against women (5.3)	Domestic	**High**
(BeLue et al., 2012)	Systems thinking tools as applied to community-based participatory research: A case study	Diagram	Increase understanding	**High**	Adolescent health, social determinants of health		Domestic	Medium
(Bernard et al., 1977)	Experimental: A simulation of the distribution of services to mentally deficient children	Tested/ Analyzed Model	Strategic planning (compare one plan to another)	**High**	Access to health services, early and middle childhood, Mental and mental disorders		Global	Low
(Brennan et al., 2015)	Systems thinking in 49 communities related to healthy eating, active living, and childhood obesity	Diagram	Increase understanding	**High**	Early and middle childhood, educational and community-based programs, health related quality and wellbeing, maternal, infant, and child health, nutrition and weight status, physical activity, social determinants of health		Domestic	**High**
(Bridgewater et al., 2011)	A community-based systems learning approach to understanding youth violence in Boston	Tested/ Analyzed Model	Increase understanding	**High**	Adolescent health, injury and violence prevention, social determinants of health	End abuse, exploitation, trafficking and all forms of violence against and torture of children (16.2)	Domestic	**High**
(Carrete et al., 2017)	A socioecological view toward an understanding of how to prevent overweight in children	Tested/ Analyzed Model	Strategic planning (compare one plan to another)	**High**	Early and middle childhood, educational and community-based programs, nutrition and weight status, physical activity, global health, social determinants of health	End preventable deaths (3.2)	Global	**High**
(Crettenden, McCarty et al. 2014)	How evidence-based workforce planning in Australia is informing policy development in the retention and distribution of the health workforce	Tested/ Analyzed Model	Strategic planning (compare one plan to another)	Low	Access to health services, Public Health Infrastructure	Access to sexual/ reproductive healthcare (3.7)	Global	Low
(Davison, Vanderwater et al. 2012)	A control-theory reward-based approach to behavior modification in the presence of social-norm pressure and conformity pressure	Tested/ Analyzed Model	Increase understanding	None	Early and middle childhood, physical activity		Global	Low
(De Silva, 2017)	How many Medical specialists do Ministry of Health- Sri Lanka need by 2025: Use of system dynamics modelling for policy decisions	Tested/ Analyzed Model	Prediction	None	Access to health services, global health, Public Health infrastructure		Global	Low
(Demir et al., 2014)	Modelling length of stay and patient flows: Methodological case studies from the UK neonatal care services	Tested/ Analyzed Model	Strategic planning (compare one plan to another)	Low	Access to health services, maternal, infant, and child health, Public Health Infrastructure		Global	**High**
(Diaz et al., 2012)	A system dynamics model for simulating ambulatory health care demands	Tested/ Analyzed Model	Prediction	None	Access to health services		Domestic	Medium
(Edelstein et al., 2015)	Reducing early childhood caries in a Medicaid population: A systems model analysis	Tested/ Analyzed Model	Strategic planning (compare one plan to another)	None	Access to health services, early and middle childhood, educational and community-based programs, maternal, infant, and child health, oral health		Domestic	**High**
(Evenden et al., 2006)	Improving the cost-effectiveness of Chlamydia screening with targeted screening strategies	Tested/ Analyzed Model	Strategic planning (compare one plan to another)	None	Access to health services, sexually transmitted diseases	End disease epidemics (3.3), Access to sexual/ reproductive healthcare (3.7)	Global	**High**
(Fallah-Fini et al., 2014)	Modeling US adult obesity trends: A system dynamics model for estimating energy imbalance gap	Tested/ Analyzed Model	Increase understanding	None	Nutrition and weight status		Domestic	**High**
(Finegood et al., 2010)	Implications of the Foresight obesity system map for solutions to childhood obesity	Diagram	Increase understanding	None	Maternal, infant, and child health, nutrition and weight status		Global	Medium
(Fredericks et al., 2008)	Using system dynamics as an evaluation tool—Experience from a demonstration program	Diagram	Increase understanding	**High**	Disability and health, educational and community-based programs		Domestic	Low
(Frerichs et al., 2013)	Modeling social transmission dynamics of unhealthy behaviors for evaluating prevention and treatment interventions on childhood obesity	Tested/ Analyzed Model	Strategic planning (compare one plan to another)	None	Nutrition and weight status		Domestic	**High**
(Frerichs et al., 2015)	Influence of school architecture and design on healthy eating: A review of the evidence	Diagram	Increase understanding	None	Early and middle childhood, maternal, infant, and child health, nutrition and weight status, social determinants of health		Both	**High**
(Frerichs, Lich, et al., 2018; Frerichs, Young, et al., 2018)	Development of a Systems Science Curriculum to Engage Rural African American Teens in Understanding and Addressing Childhood Obesity Prevention	Diagram	Increase understanding	**High**	Adolescent health, nutrition and weight status, social determinants of health, physical activity	End preventable deaths 3.2)	Domestic	**High**
(Frerichs, Lich, et al., 2018; Frerichs, Young, et al., 2018)	Mind maps and network analysis to evaluate conceptualization of complex issues: A case example evaluating systems science workshops for childhood obesity prevention	Concept model, untested	Increase understanding	**High**	Adolescent health, nutrition and weight status, physical activity, social determinants of health	End preventable deaths (3.2)	Domestic	Low
(Ghaffarzadegan et al., 2013)	Practice variation, bias, and experiential learning in Cesarean delivery: A data-based system dynamics approach	Tested/ Analyzed Model	Increase understanding	None	Maternal, infant, and child health		Domestic	Low
(Gillen et al., 2014)	Social ecology of asthma: Engaging stakeholders in integrating health behavior theories and practice-based evidence through systems mapping	Diagram	Increase understanding	**High**	Early and middle childhood, health communication and health information technology, respiratory diseases		Domestic	**High**
(Goncalves & Kamdem, 2016)	Reaching an AIDS-Free Generation in Cote d'Ivoire, Data Driven Policy Design for HIV/AIDS Response Programs: Evidence-Based Policy Design for HIV/AIDS Response Programs in Cote d'Ivoire	Tested/ Analyzed Model	Informing policy	None	Global health, HIV, immunization and infectious disease, sexually transmitted diseases, maternal, infant, and child health	End preventable deaths (3.2), End disease epidemics (3.3), Access to sexual/reproductive healthcare (3.7)	Global	Low
(Grove, 2015)	Aiming for utility in 'systems-based evaluation': A research-based framework for practitioners	Concept model, untested	Increase understanding	**High**	Access to health services, global health, sexually transmitted diseases	End disease epidemics (3.3)	Global	Low
(Hamdani et al., 2011)	Systems thinking perspectives applied to healthcare transition for youth with disabilities: A paradigm shift for practice, policy and research	Diagram	Increase understanding	None	Access to health services, adolescent health, Disability and health		Both	Medium
(Lich et al., 2017)	Extending systems thinking in planning and evaluation using group concept mapping and system dynamics to tackle complex problems	Concept model, untested	Increase understanding	**High**	Adolescent health, mental health and mental disorders, disability and health, health related quality of life and wellbeing		Domestic	**High**
(Heidenberger & Flessa, 1993)	A system dynamics model for AIDS policy support in Tanzania	Tested/ Analyzed Model	Increase understanding	Low	HIV, sexually transmitted diseases	End disease epidemics (3.3)	Global	Medium
(Hernandez et al., 2016)	Enhancing Antenatal Clinics Decision-Making Through the Modelling and Simulation of Patients Flow by Using a System Dynamics Approach. A Case for a British Northwest Hospital	Tested/ Analyzed Model	Strategic planning (compare one plan to another)	Low	Access to health services, global health	Reduce maternal mortality (3.1), Access to sexual/reproductive healthcare (3.7)	Global	Low
(Hirsch et al., 2012)	A Simulation model for designing effective interventions in early childhood caries	Tested/ Analyzed Model	Strategic planning (compare one plan to another)	Low	Early and middle childhood, educational and community-based programs, oral health		Domestic	**High**
(Hoehner et al., 2015)	Behavior-over-time graphs: Assessing perceived trends in healthy eating and active living environments and behaviors across 49 communities	Diagram	Increase understanding	**High**	Early and middle childhood, educational and community-based programs, health related quality of life and wellbeing, maternal, infant, and child health, nutrition and weight status, physical activity, social determinants of health		Domestic	**High**
(Holder & Blose, 1987)	Reduction of community alcohol-problems: Computer simulation experiments in 3 countries	Tested/ Analyzed Model	Strategic planning (compare one plan to another)	None	Substance abuse	Strengthen prevention and treatment of substance abuse (3.5)	Domestic	Medium
(Hontelez et al., 2016)	Changing HIV treatment eligibility under health system constraints in sub-Saharan Africa: investment needs, population health gains, and cost-effectiveness	Tested/ Analyzed Model	Strategic planning (compare one plan to another)	None	Access to health services, global health, HIV, sexually transmitted diseases, immunization and infectious disease	End preventable deaths (3.2), End disease epidemics (3.3), Access to sexual/reproductive healthcare (3.7)	Global	Low
(Hosseinichimeh et al., 2018)	Modeling and estimating the feedback mechanisms among depression, rumination, and stressors in adolescents	Tested/ Analyzed Model	Increase understanding	**High**	Adolescent health, mental and mental disorders		Domestic	**High**
(Hovmand & Ford, 2009)	Sequence and timing of three community interventions to domestic violence	Tested/ Analyzed Model	Increase understanding	**High**	Injury and violence prevention	Eliminate violence against women (5.2)	Domestic	**High**
(Hovmand et al., 2009)	Victims arrested for domestic violence: Unintended consequences of arrest policies	Tested/ Analyzed Model	Increase understanding	**High**	Injury and violence prevention	Eliminate violence against women (5.2)	Domestic	**High**
(Huang et al., 2013)	Epidemiology of Kawasaki disease: Prevalence from national database and future trends projection by system dynamics modeling	Tested/ Analyzed Model	Prediction	None	Maternal, infant, and child health		Both	Low
(Ishikawa, Ohba et al. 2013)	Forecasting the absolute and relative shortage of physicians in Japan using a system dynamics model approach	Tested/ Analyzed Model	Prediction	None	Access to health services	Access to sexual/ reproductive healthcare (3.7)	Global	Low
(Jalali et al., 2017)	Dynamics of Implementation and Maintenance of Organizational Health Interventions	Diagram	Increase understanding	**High**	Early and middle childhood, nutrition and weight status, physical activity, educational and community-based programs		Domestic	**High**
(Keane et al., 2015)	Healthy Kids, Healthy Cuba: Findings From a group model building process in the rural Southwest	Diagram	Increase understanding	**High**	Early and middle childhood, educational and community-based programs, health related quality of life and wellbeing, maternal, infant, and child health, nutrition and weight status, physical activity, social determinants of health		Domestic	**High**
(Kok et al., 2015)	Optimizing an HIV testing program using a system dynamics model of the continuum of care	Tested/Analyzed Model	Strategic planning (compare one plan to another)	**High**	Access to health services, global health, HIV, lesbian, gay, bisexual, and transgender health, Public Health Infrastructure, sexually transmitted diseases	End disease epidemics (3.3)	Global	**High**
(Kommer, 2002)	A waiting list model for residential care for the mentally disabled in The Netherlands	Tested/ Analyzed Model	Informing policy	**High**	Mental and mental disorders		Global	Low
(Kumar & Kumar, 2014)	Modelling rural healthcare supply chain in India using system dynamics	Concept model, untested	Increase understanding	Low	Access to health services, maternal, infant, and child health, Public Health Infrastructure	End preventable deaths (3.2)	Global	Low
(Lan, Chen et al. 2014)	An Investigation of Factors Affecting Elementary School Students' BMI Values Based on the System Dynamics Modeling	Tested/ Analyzed Model	Increase understanding	None	Early and middle childhood, nutrition and weight status		Global	Medium
(Lee et al., 2016)	A system dynamics modelling approach to studying the increasing prevalence of people with intellectual developmental disorders in New South Wales	Tested/Analyzed Model	Prediction	None	Disability and health, global health, mental and mental disorders, early and middle childhood		Global	Low
(Liu et al., 2016)	Systems simulation model for assessing the sustainability and synergistic impacts of sugar-sweetened beverages tax and revenue recycling on childhood obesity prevention	Tested/ Analyzed Model	Informing policy	None	Early and middle childhood, nutrition and weight status, physical activity, social determinants of health, adolescent health		Domestic	**High**
(Lounsbury et al., 2015)	Simulating patterns of patient engagement, treatment adherence, and viral suppression: A system dynamics approach to evaluating HIV care management	Tested/ Analyzed Model	Increase understanding	None	HIV, sexually transmitted diseases	End disease epidemics (3.3)	Domestic	**High**
(Lyon et al., 2016)	Modeling the impact of school-based universal depression screening on additional service capacity needs: A system dynamics approach	Concept model, untested	Strategic planning (compare one plan to another)	None	Access to health services, adolescent health, educational and community-based programs, Mental and mental disorders		Domestic	**High**
(Maital & Bornstein, 2003)	The ecology of collaborative child rearing: A systems approach to child care on the kibbutz	Diagram	Increase understanding	None	Early and middle childhood		Global	Low
(Martin et al., 2015a, 2015b, 2015c)	Modeling the declining positivity rates for Human Immunodeficiency Virus testing in New York state	Tested/ Analyzed Model	Increase understanding	None	HIV, sexually transmitted diseases	End disease epidemics (3.3)	Domestic	**High**
(Martin et al., 2015a, 2015b, 2015c)	Policy modeling to support administrative decision making on the New York state HIV testing law	Tested/Analyzed Model	Informing policy	Low	HIV, sexually transmitted diseases	End disease epidemics (3.3)	Domestic	Low
(Martin et al., 2015a, 2015b, 2015c)	Mandating the offer of HIV testing in New York: Simulating the epidemic impact and resource needs	Concept model, untested	Informing policy	**High**	HIV, sexually transmitted diseases	End disease epidemics (3.3)	Domestic	Low
(McGlashan et al., 2016)	Quantifying a Systems Map: Network Analysis of a Childhood Obesity Causal Loop Diagram	Diagram	Increase understanding	**High**	Early and middle childhood, global health, nutrition and weight status, physical activity, social determinants of health		Global	Low
(McKibben et al., 2016)	Projecting the urology workforce over the next 20 years	Concept model, untested	Prediction	None	Access to health services, family planning	Access to sexual/reproductive healthcare (3.7)	Domestic	Low
(Meisel et al., 2018)	Towards a novel model for studying the nutritional stage dynamics of the Colombian population by age and socioeconomic status	Tested/Analyzed Model	Prediction	None	Adolescent health, early and middle childhood, global health, nutrition and weight status, physical activity	End preventable deaths (3.2)	Global	Low
(Minyard et al., 2014)	Using systems thinking in state health policymaking: an educational initiative	Concept model, untested	Increase understanding	**High**	Health communication and health information technology, nutrition and weight status		Domestic	**High**
(Moreland, 2015)	Improving park space access for the Healthy Kids, Healthy Communities Partnership in Denver, Colorado	Diagram	Increase understanding	**High**	Early and middle childhood, nutrition and weight status, physical activity, social determinants of health		Domestic	**High**
(Moxnes & Jensen, 2009)	Drunker than intended: Misperceptions and information treatments	Tested/ Analyzed Model	Increase understanding	None	Adolescent health, substance abuse	Strengthen prevention and treatment of substance abuse (3.5)	Global	Medium
(Munar et al., 2015)	Scaling-up impact in perinatology through systems science: Bridging the collaboration and translational divides in cross-disciplinary research and public policy	Diagram	Increase understanding	**High**	Access to health services, global health, maternal, infant, and child health	Reduce maternal mortality (3.1), End preventable deaths (3.2)	Global	**High**
(Nadkarni et al., 2018)	Modeling patient access to therapeutic oxytocin in Zanzibar, Tanzania	Tested/ Analyzed Model	Prediction	None	Access to health services, global health, maternal, infant, and child health, Public Health infrastructure	Reduce maternal mortality (3.1), End preventable deaths (3.2), Access to sexual/reproductive healthcare (3.7)	Global	Low
(Nelson et al., 2015)	Using group model building to understand factors That influence childhood obesity in an urban environment	Diagram	Increase understanding	**High**	Educational and community-based programs, nutrition and weight status, physical activity, social determinants of health		Domestic	**High**
(Osgood, Dyck, et al., 2011; Osgood, Mahamoud, et al., 2011)	The Inter- and Intragenerational Impact of Gestational Diabetes on the Epidemic of Type 2 Diabetes	Tested/ Analyzed Model	Increase understanding	None	Diabetes, maternal, infant, and child health		Global	Medium
(Osgood, Dyck, et al., 2011; Osgood, Mahamoud, et al., 2011)	Estimating the relative impact of early-life infection exposure on later-life tuberculosis outcomes in a Canadian sample	Tested/ Analyzed Model	Increase understanding	None	Immunization and infectious disease, respiratory diseases, social determinants of health	End disease epidemics (3.3)	Global	Medium
(Owen et al., 2018)	Understanding a successful obesity prevention initiative in children under 5 from a systems perspective	Diagram	Increase understanding	**High**	Early and middle childhood, nutrition and weight status, physical activity	End preventable deaths (3.2)	Global	Low
(Ozawa et al., 2016)	Exploring pathways for building trust in vaccination and strengthening health system resilience	Diagram	Increase understanding	None	Public Health infrastructure, immunization and infectious disease, maternal, infant, and child health, health communication and health information technology	End preventable deaths (3.2), End disease epidemics (3.3)	Global	**High**
(Page et al., 2017)	A decision-support tool to inform Australian strategies for preventing suicide and suicidal behaviour	Tested/ Analyzed Model	Strategic planning (compare one plan to another)	None	Health related quality of life, mental and mental disorders, injury and violence	End preventable deaths (3.2)	Global	Low
Patil MK, Janahanlal PS.(Patil & Janahanlal, 1978)	A system dynamics feedback control model study of population of "India 2001" and policies for stabilizing growth	Tested/ Analyzed Model	Informing policy	None	Family planning, global health		Global	Low
Patrick H, Hennessy E, McSpadden K, Oh A.(Patrick et al., 2013)	Parenting styles and practices in children's obesogenic behaviors: Scientific gaps and future research directions	Diagram	Increase understanding	None	Early and middle childhood, nutrition and weight status		Domestic	Low
Pedamallu CS, Ozdamar L, Kropat E, Weber GW.(Pedamallu et al., 2012)	A system dynamics model for intentional transmission of HIV/AIDS using cross impact analysis	Tested/ Analyzed Model	Strategic planning (compare one plan to another)	None	HIV, sexually transmitted diseases	End disease epidemics (3.3)	Both	**High**
Pieters A, Akkermans H, Franx A.(Pieters et al., 2011)	E pluribus unum: Using group model building with many interdependent organizations to create integrated health care networks	Diagram	Strategic planning (compare one plan to another)	**High**	Access to health services, maternal, infant, and child health	Reduce maternal mortality (3.1), Access to sexual/ reproductive healthcare (3.7)	Global	Low
Pieters, A.; van Oorschot, K. E.; Akkermans, H. A.; Brailsford, S. C.(Pieters, van Oorschot et al. 2018)	Improving inter-organizational care-cure designs: specialization versus integration	Tested/ Analyzed Model	Strategic planning (compare one plan to another)	**High**	Access to health services, maternal, infant, and child health, Public Health infrastructure	Reduce maternal mortality (3.1), Access to sexual/reproductive healthcare (3.7)	Global	**High**
Powell, K. E.; Kibbe, D. L.; Ferencik, R.; Soderquist, C.; Phillips, M. A.; Vall, E. A.; Minyard, K. J.(Powell et al., 2017)	Systems Thinking and Simulation Modeling to Inform Childhood Obesity Policy and Practice	Concept model, untested	Informing policy	**High**	Adolescent health, early and middle childhood, educational and community-based programs, nutrition and weight status, physical activity		Domestic	**High**
Rauner MS.(Rauner, 2002)	Resource allocation for HIV/AIDS control programs: a model-based policy analysis	Tested/ Analyzed Model	Increase understanding	None	HIV, sexually transmitted diseases	End disease epidemics (3.3)	Global	Low
Rosas, S. R.(Rosas, 2017)	Systems thinking and complexity: considerations for health promoting schools	Concept model, untested	Increase understanding	None	Adolescent health, early and middle childhood, educational and community-based programs, social determinants of health, nutrition and weight status, physical activity, health related quality of life and wellbeing		Domestic	Low
(Rwashana, Nakubulwa et al. 2014)	Advancing the application of systems thinking in health: understanding the dynamics of neonatal mortality in Uganda	Diagram	Increase understanding	**High**	Maternal, infant, and child health	End preventable deaths (3.2)	Global	**High**
(Rwashana et al., 2009)	System dynamics approach to immunization healthcare issues in developing countries: a case study of Uganda	Diagram	Increase understanding	**High**	Early and middle childhood, immunization and infectious disease	End preventable deaths (3.2), End disease epidemics (3.3)	Global	**High**
(Sabounchi et al., 2014)	A novel system dynamics model of female obesity and fertility	Tested/ Analyzed Model	Increase understanding	None	Maternal, infant, and child health, nutrition and weight status		Domestic	**High**
(Schrottner, Konig et al. 2009)	A population prospect for future health care models based on a system dynamics model	Tested/ Analyzed Model	Increase understanding	None			Global	Low
(Schuh et al., 2017)	Examining the structure and behavior of Afghanistan's routine childhood immunization system using system dynamics modeling	Tested/ Analyzed Model	Increase understanding	None	Early and middle childhood, global health, immunization and infectious disease, maternal, infant, and child health, access to health services, Public Health infrastructure	End preventable deaths (3.2), End disease epidemics (3.3)	Global	Low
(Semwanga et al., 2016)	Applying a system dynamics modelling approach to explore policy options for improving neonatal health in Uganda	Tested/Analyzed Model	Strategic planning (compare one plan to another)	**High**	Access to health services, educational and community-based programs, global health, maternal, infant, and child health	Reduce maternal mortality (3.1), End preventable deaths (3.2), Access to sexual/reproductive healthcare (3.7)	Global	**High**
(Shariatpanahi et al., 2017)	Assessing the effectiveness of disease awareness programs: Evidence from Google Trends data for the world awareness dates	Tested/ Analyzed Model	Increase understanding	None	Educational and community-based programs, global health	End preventable deaths (3.2)	Both	Low
(Sheldrick et al., 2016)	A system dynamics model of clinical decision thresholds for the detection of developmental-behavioral disorders	Tested/ Analyzed Model	Strategic planning (compare one plan to another)	None	Early and middle childhood, disability and health, hearing and other sensory or communication disorders, mental and mental disorders		Domestic	Low
(Siegel et al., 2011)	Real-time tool to display the predicted disease course and treatment response for children with Crohn's disease	Tested/ Analyzed Model	Increase understanding	None	Early and middle childhood, health communication and health information technology		Domestic	Low
(Soler et al., 2016)	Community-Based Interventions to Decrease Obesity and Tobacco Exposure and Reduce Health Care Costs: Outcome Estimates From Communities Putting Prevention to Work for 2010–2020	Tested/ Analyzed Model	Strategic planning (compare one plan to another)	None	Educational and community-based programs, nutrition and weight status, physical activity, tobacco use, heart disease and stroke	End preventable deaths (3.2)	Domestic	Low
(Staller, 2004)	Runaway youth system dynamics: A theoretical framework for analyzing runaway and homeless youth policy	Diagram	Increase understanding	None	Adolescent health, social determinants of health		Domestic	Low
(Tebbens & Thompson, 2018)	Using integrated modeling to support the global eradication of vaccine-preventable diseases	Tested/ Analyzed Model	Increase understanding	None	Global health, immunization and infectious disease, maternal, infant, and child health, early and middle childhood	End preventable deaths (3.2), End disease epidemics (3.3)	Global	Low
(Tengs et al., 2001)	The cost-effectiveness of intensive national school-based anti-tobacco education: Results from the Tobacco Policy Model	Tested/ Analyzed Model	Strategic planning (compare one plan to another)	None	Adolescent health, health related quality of life and wellbeing, substance abuse, tobacco use	Strengthen prevention and treatment of substance abuse (3.5)	Domestic	Medium
(Thomas & Reilly, 2015)	Group model building: A framework for organizing healthy community program and policy initiatives in Columbia, Missouri	Diagram	Increase understanding	**High**	Early and middle childhood, health communication and health information technology, nutrition and weight status, physical activity, social determinants of health		Domestic	**High**
(Townshend & Turner, 2000)	Analysing the effectiveness of Chlamydia screening	Tested/ Analyzed Model	Strategic planning (compare one plan to another)	Low	Maternal, infant, and child health, sexually transmitted diseases	End disease epidemics (3.3), Access to sexual/ reproductive healthcare (3.7)	Global	Medium
(Viana et al., 2014)	Combining discrete-event simulation and system dynamics in a healthcare setting: A composite model for Chlamydia infection	Tested/ Analyzed Model	Strategic planning (compare one plan to another)	**High**	Access to health services, sexually transmitted diseases	End disease epidemics (3.3), Access to sexual/ reproductive healthcare (3.7)	Global	Medium
(Weeks et al., 2013)	Multilevel dynamic systems affecting introduction of HIV/STI prevention innovations among Chinese women in sex work establishments	Diagram	Increase understanding	**High**	Educational and community-based programs, HIV, sexually transmitted diseases	End disease epidemics (3.3), Access to sexual reproductive health and rights (5.6)	Global	**High**
(Weeks et al., 2017)	Using Participatory System Dynamics Modeling to Examine the Local HIV Test and Treatment Care Continuum in Order to Reduce Community Viral Load	Diagram	Increase understanding	**High**	Immunization and infectious disease, HIV, sexually transmitted diseases	End preventable deaths (3.2), End disease epidemics (3.3)	Domestic	Low
(Wu et al., 2013)	Theoretical system dynamics modeling for Taiwan pediatric workforce in an era of national health insurance and low birth rates	Tested/ Analyzed Model	Prediction	None	Access to health services, adolescent health, early and middle childhood, maternal, infant, and child health, Public Health Infrastructure	End preventable deaths (3.2)	Global	Low
(Yourkavitch et al., 2018)	Interactions among poverty, gender, and health systems affect women's participation in services to prevent HIV transmission from mother to child: A causal loop analysis	Diagram	Increase understanding	**High**	Social determinants of health, health related quality of life and wellbeing, maternal, infant, and child health, sexually transmitted diseases, HIV, immunization and infectious disease, educational and community-based programs	End preventable deaths (3.2), End disease epidemics (3.3), Access to sexual/reproductive healthcare (3.7)	Global	Low
(Zou et al., 2018)	Strategies to control HIV and HCV in methadone maintenance treatment in Guangdong Province, China: a system dynamic modeling study	Tested/Analyzed Model	Strategic planning (compare one plan to another)	None	Global health, HIV, sexually transmitted diseases, immunization and infectious disease, access to health services, substance abuse	End preventable deaths (3.2), End disease epidemics (3.3), Strengthen prevention and treatment of substance abuse (3.5), Access to sexual/reproductive healthcare (3.7)	Global	Low

*SD approach* was classified using the following definitions: qualitative diagrams were defined as a causal loop diagram and/or stock and flow diagram created to better understand complex, problematic trends without using numbers and data, concept models were defined as a SD model informed by preliminary (or hypothetical) numbers that is used to test hypotheses and explore effects of feedback in a system, and tested/analyzed models were defined as a calibrated/validated SD model used to generate evidence via simulation modeling

*Model purpose* was classified using the following definitions: increase understanding was defined as using a model to increase scientific understanding, strategic planning was defined as using a model to compare the effectiveness of interventions or policies to inform decision-making, informing policy was defined as using a model to answer questions related to a specific existing or proposed policy, and predicting was defined as using a model to project future system behavior based on past system behavior

*Level of stakeholder engagement* was classified using the following criteria: none was defined as complete absence of any description of stakeholder engagement, low was defined as single encounters with stakeholders that were siloed from larger modeling process, and high was defined as stakeholders actively engaged in the mapping or modeling process

*Utility for MCH policy/practice* was a subjectively scored measure reflecting the extent to which the three MCH experts believe the article is particularly useful in informing future policy/practice initiatives on related topics. Together, these three experts have over 80 years of professional experience in the MCH field

## Results

**Fig. 3:**
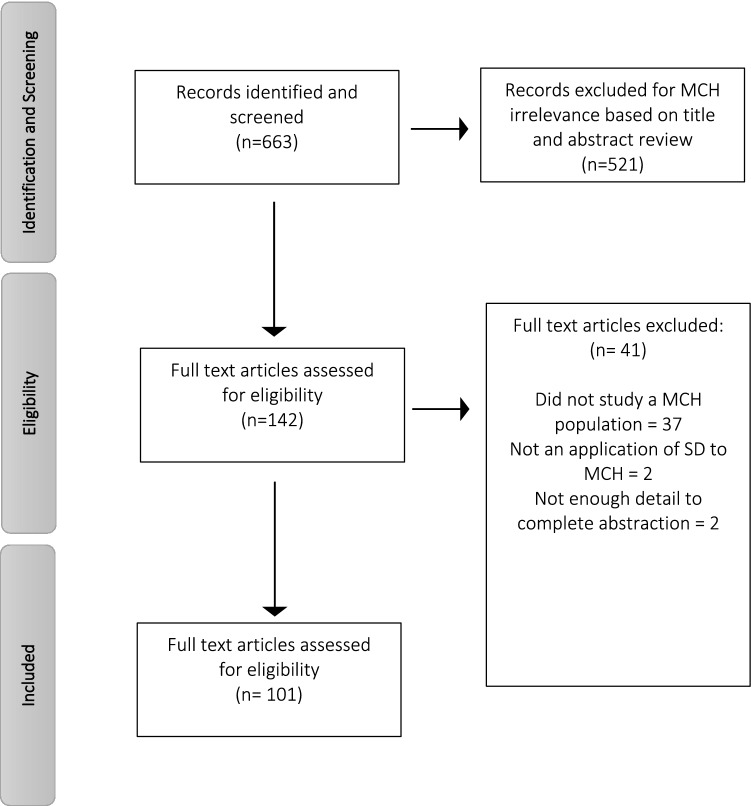
Results from Step 4 of Search Strategy (Moher et al., 2009)

*Steps 1–3* identified 663 articles meeting criteria of SD methods applied to health. Of those articles, 521 articles were excluded for MCH irrelevance based on title and abstract review (Fig. 3). Additionally, 41 articles were excluded after full text review because they did not study an MCH population (n = 37), they were not an application of SD to MCH (n = 2), or the description of the SD work was not detailed enough to permit abstraction of the relevant information (n = 2). A total of 101 articles met all inclusion criteria and were included in this review (Table 1).

### SD Approach

Of the 101 included works, by far the most common SD approach described was tested/analyzed models (n = 67). One example was in Hosseinichimeh et al., (2018), where the authors built, tested, and analyzed a SD model (Fig. 2e) to holistically study the complex relationships among stressors, rumination, and depression. Longitudinal, primary data collected from middle-school students was used an input data to simulate evidence on impact of prior stressors on current levels of depression for an adolescent population (Fig. 2f). This SD approach allowed researchers to better understand feedback created between stressors, rumination and depression, including average time adolescents tend to ruminate after activated by a stressor and corresponding levels of depression associated with lengths of rumination. Findings indicate opportunities to improve clinical interventions targeting pediatric depression. As a second example, in Frerichs et al. (2013), the researchers compared 15 different combinations of interventions to prevent and treat childhood obesity and 6 variations on adult-to-child impact factor ratios for these interventions to identify those with the highest levels of impact over a 10-year time horizon. This paper is a compelling example of the power of a rigorously tested model to deliver insights useful for MCH decision makers.

Of the 101 included works, 27 developed qualitative diagrams. For example, in Munar, et al. (2015) a causal loop diagram (Fig. 2a) and a stock and flow diagram (Fig. 2b) were used to facilitate conversations among stakeholders participating in the Salud Mesoamerica 2015 initiative in Honduras regarding the impact of limited staff and clinic capacity on the number of children vaccinated. Authors note that qualitative SD diagrams provide “tangible” tools that help diverse stakeholders with diverse perspectives articulate complex problems; using such diagrams to guide difficult conversations shifts the focus “from whether one person is right and the other is wrong, to a discussion about whether or not the diagram is correct, captures the relevant relationships, resolves a conflict, and so on” (Munar, et al., 2015). Other topics explored using qualitative diagrams included pediatric asthma management (Gillen et al., 2014), care transitions for children with disabilities (Hamdani et al., 2011), child care (Maital & Bornstein, 2003), neonatal mortality (Rwashana, Nakubulwa et al. 2014), homeless youth policies (Staller, 2004), cross-disciplinary collaboration (Munar et al., 2015; Pieters et al., 2011), and a new implementation evaluation method for programs with complex networks of structures and stakeholders (Fredericks et al., 2008).

Finally, we found 10 examples of concept models. One exemplary instance is a teaching model created for working with policymakers from the state of Georgia on childhood obesity. (Minyard et al., 2014) Georgia policymakers chose the topic of the model, directed the project, and were led through a “learning lab” that allowed them to experiment in the model with a number of different strategies to prevent and reduce childhood obesity (Fig. 2c, 2d). The group of policymakers reported that the learning lab informed the passage of a bill that proposed a unique combination of interventions to prevent childhood obesity. While we only found 10 examples of concept models applied to MCH, we believe concept models offer valuable opportunities for the MCH workforce to engage diverse stakeholders to understand and address complex MCH problems.

### SD Purpose

We posited 4 purposes for which SD tools and approaches are utilized: increasing understanding, strategic planning, informing policy, or predicting (Table 2).

**Table 2:** Purpose of the system dynamics models

Model purpose	# of Articles	Article Citations
Increasing understanding (*Defined as using a model to increase scientific understanding of a given topic*)	55	(Heidenberger & Flessa, 1993, Rauner, 2002, Maital & Bornstein, 2003, Staller, 2004, Fredericks et al., 2008, Hovmand & Ford, 2009, Hovmand et al., 2009, Moxnes & Jensen, 2009, Rwashana et al., 2009, Schrottner, Konig et al. 2009, Finegood et al., 2010, Bridgewater et al., 2011, Hamdani et al., 2011, Osgood, Dyck, et al., 2011; Osgood, Mahamoud, et al., 2011, Osgood, Dyck, et al., 2011; Osgood, Mahamoud, et al., 2011, Siegel et al., 2011, BeLue et al., 2012, Davison, Vanderwater et al. 2012, Ghaffarzadegan et al., 2013, Patrick et al., 2013, Weeks et al., 2013, Fallah-Fini et al., 2014, Gillen et al., 2014, Kumar & Kumar, 2014, Lan, Chen et al. 2014, Minyard et al., 2014, Rwashana, Nakubulwa et al. 2014, Sabounchi et al., 2014, Batchelder et al., 2015, Brennan et al., 2015, Frerichs et al., 2015, Grove, 2015, Hoehner et al., 2015, Keane et al., 2015, Lounsbury et al., 2015, Martin et al., 2015a, 2015b, 2015c, Moreland, 2015, Munar et al., 2015, Nelson et al., 2015, Thomas & Reilly, 2015, Batchelder & Lounsbury, 2016, McGlashan et al., 2016, Ozawa et al., 2016, Jalali et al., 2017, Lich et al., 2017, Rosas, 2017, Schuh et al., 2017, Shariatpanahi et al., 2017, Weeks et al., 2017, Frerichs, Lich, et al., 2018; Frerichs, Young, et al., 2018, Frerichs, Lich, et al., 2018; Frerichs, Young, et al., 2018, Hosseinichimeh et al., 2018, Owen et al., 2018, Tebbens & Thompson, 2018, Yourkavitch et al., 2018)
Strategic Planning *(Defined as using a model to compare the effectiveness of interventions or policies to inform decision-making)*	26	(Bernard et al., 1977, Holder & Blose, 1987, Anderson & Anderson, 1998, Townshend & Turner, 2000, Tengs et al., 2001, Evenden et al., 2006, Barber & Lopez-Valcarcel, 2010, Pieters et al., 2011, Hirsch et al., 2012, Pedamallu et al., 2012, Frerichs et al., 2013, Crettenden, McCarty et al. 2014, Demir et al., 2014, Viana et al., 2014, Edelstein et al., 2015, Kok et al., 2015, Hernandez et al., 2016, Hontelez et al., 2016, Lyon et al., 2016, Semwanga et al., 2016, Sheldrick et al., 2016, Soler et al., 2016, Carrete et al., 2017, Page et al., 2017, Pieters, van Oorschot et al. 2018, Zou et al., 2018)
Informing policy *(Defined as using a model to answer questions related to a specific existing or proposed policy)*	11	(Patil & Janahanlal, 1978, Kommer, 2002, Ahmad, 2005a, 2005b, Ahmad, 2005a, 2005b, Ahmad & Billimek, 2007, Abidin, Mamat et al. 2014, Martin et al., 2015a, 2015b, 2015c, Martin et al., 2015a, 2015b, 2015c, Goncalves & Kamdem, 2016, Liu et al., 2016, Powell et al., 2017)
Predicting *(Defined as using a model to project future system behavior based on past system behavior)*	9	(Diaz et al., 2012, Huang et al., 2013, Ishikawa, Ohba et al. 2013, Wu et al., 2013, Lee et al., 2016, McKibben et al., 2016, De Silva, 2017, Meisel et al., 2018, Nadkarni et al., 2018)

The most common model purpose we identified was increasing understanding (~ 55% of results). One example, by Moxnes and Jensen (2009), describes the creation of a tested/analyzed model that simulates the user’s blood alcohol concentration (BAC). This model was used by high school students to explore a number of scenarios where teens exceed their intended BAC: drinking with a full stomach, and drinking to attain a particular level of BAC. When compared to students who received written educational materials, students who used the simulation to experiment with different drinking behaviors were better able to learn lessons that might help them avoid future binge drinking. This and another study (Siegel et al., 2011) show a promising use of SD models in helping individuals increase their understanding in order to modify risky behaviors after experimentation in a “learning lab” environment. Another example is Osgood, Dyck, et al. (2011), Osgood, Mahamoud, et al. (2011)), which studied the impact of gestational diabetes on future risk of developing type-2 diabetes for women and their children. Using data from Saskatchewan, they used a tested/analyzed model to trace the population’s progress through different disease states. The second example is more typical of SD models that attempt to increase scientific understanding.

The second most common model purpose identified was strategic planning, which involves comparing effectiveness among interventions or policies to inform decision-making (~ 25% of results). Hirsch et al. (2012) used this type of model to compare the costs and effectiveness of six different types of interventions addressing early childhood caries (tooth decay) singly and in combination. The authors used a tested/analyzed model with a ten-year time horizon, and used national and state data from the Colorado Child Health Survey, the National Health and Nutrition Examination Survey and the Medical Expenditures Panel Survey to make these results Colorado specific.

The third most common model purpose identified was informing policy (~ 11% of results). An example of this type of work is found in Ahmad’s articles (2005 & 2007) on tobacco policies (see Table 1), one of which compared the effects of a United States legal smoking age of 21 versus 18 (Ahmad, 2005a, 2005b). Using a tested/analyzed model with a 50-year time horizon, the author examines three different scenarios for how smoking behaviors (and subsequent health and cost outcomes) might be affected. The input values for the model came from national surveys and the literature, and were tested in a sensitivity analysis.

The final modeling purpose is predicting, where the researcher uses past system behavior to project future system behavior. Nine works (~ 9%) created a model for this purpose. These were used to predict ambulatory health care demand (Diaz et al., 2012), the US urology workforce (McKibben et al., 2016), the prevalence of people with intellectual developmental disorders (Lee et al., 2016), the prevalence of Kawasaki disease (Huang et al., 2013), the Taiwanese pediatric workforce (Wu et al., 2013), the shortage of physicians in Japan (Ishikawa et al. 2013), medical specialists needed in Sri Lanka (De Silva, 2017), nutrition status of the Colombian population (Meisel et al., 2018), and the supply of therapeutic oxytocin in Tanzania (Nadkarni et al., 2018).

### MCH Topics

The content abstraction identified a broad range of Healthy People 2020 objectives studied (Table 3). While the topics studied in the 101 works varied, the two Healthy People 2020 objectives addressed most frequently were early and middle childhood, addressed by ~ 30% of the studies, and access to health services, addressed by ~ 26% (Table 3); the topic of early and middle childhood seeks to improve the healthy development, health, safety, and well-being of adolescents and young adults, and the topic of access to health services seeks to improve access to comprehensive, quality health care services. An example of studying the topic of early and middle childhood was found in Liu et al. (2016), where a tested/analyzed model compared three possible interventions to implement a tax on sugar-sweetened beverage to understand the impact on children’s weight over time. Of the works identified focusing on early and middle childhood, obesity and nutrition was most studied. Other examples of early and middle childhood literature included studies on developmental disorders (Bernard et al., 1977; Lee et al., 2016; Sheldrick et al., 2016) and immunization (Rwashana et al., 2009; Schuh et al., 2017). Of those works addressing access to health services literature, several specifically spoke to workforce needs (Barber & Lopez-Valcarcel, 2010, Ishikawa et al., 2013, Wu et al., 2013, Crettenden, McCarty et al. 2014, McKibben et al., 2016, De Silva, 2017) and STI-related services (Evenden et al., 2006; Hontelez et al., 2016; Kok et al., 2015; Viana et al., 2014; Zou et al., 2018).

**Table 3:** Healthy people 2020 goals

Healthy people 2020 goal	# of Articles	Article citations
Access to Health Services	26	(Bernard et al., 1977, Evenden et al., 2006, Barber & Lopez-Valcarcel, 2010, Hamdani et al., 2011, Pieters et al., 2011, Diaz et al., 2012, Ishikawa, Ohba et al. 2013, Wu et al., 2013, Crettenden, McCarty et al. 2014, Demir et al., 2014, Kumar & Kumar, 2014, Viana et al., 2014, Edelstein et al., 2015, Grove, 2015, Kok et al., 2015, Munar et al., 2015, Hernandez et al., 2016, Hontelez et al., 2016, Lyon et al., 2016, McKibben et al., 2016, Semwanga et al., 2016, De Silva, 2017, Schuh et al., 2017, Nadkarni et al., 2018, Pieters, van Oorschot et al. 2018, Zou et al., 2018)
Adolescent Health	20	(Tengs et al., 2001, Staller, 2004, Ahmad, 2005a, 2005b, Ahmad, 2005a, 2005b, Ahmad & Billimek, 2007, Moxnes & Jensen, 2009, Bridgewater et al., 2011, Hamdani et al., 2011, BeLue et al., 2012, Wu et al., 2013, Abidin, Mamat et al. 2014, Liu et al., 2016, Lyon et al., 2016, Lich et al., 2017, Powell et al., 2017, Rosas, 2017, Frerichs, Lich, et al., 2018; Frerichs, Young, et al., 2018, Frerichs, Lich, et al., 2018; Frerichs, Young, et al., 2018, Hosseinichimeh et al., 2018, Meisel et al., 2018)
Cancer	0	None
Diabetes	1	(Osgood, Dyck, et al., 2011; Osgood, Mahamoud, et al., 2011)
Disability and Health	5	(Fredericks et al., 2008; Hamdani et al., 2011; Lee et al., 2016; Lich et al., 2017; Sheldrick et al., 2016)
Early and Middle Childhood	30	(Bernard et al., 1977, Maital & Bornstein, 2003, Rwashana et al., 2009, Siegel et al., 2011, Davison, Vanderwater et al. 2012, Hirsch et al., 2012, Patrick et al., 2013, Wu et al., 2013, Abidin, Mamat et al. 2014, Gillen et al., 2014, Lan, Chen et al. 2014, Brennan et al., 2015, Edelstein et al., 2015, Frerichs et al., 2015, Hoehner et al., 2015, Keane et al., 2015, Moreland, 2015, Thomas & Reilly, 2015, Lee et al., 2016, Liu et al., 2016, McGlashan et al., 2016, Sheldrick et al., 2016, Carrete et al., 2017, Jalali et al., 2017, Powell et al., 2017, Rosas, 2017, Schuh et al., 2017, Meisel et al., 2018, Owen et al., 2018, Tebbens & Thompson, 2018)
Educational and Community-Based Programs	17	(Brennan et al., 2015; Carrete et al., 2017; Edelstein et al., 2015; Fredericks et al., 2008; Hirsch et al., 2012; Hoehner et al., 2015; Jalali et al., 2017; Keane et al., 2015; Lyon et al., 2016; Nelson et al., 2015; Powell et al., 2017; Rosas, 2017; Semwanga et al., 2016; Shariatpanahi et al., 2017; Soler et al., 2016; Weeks et al., 2013; Yourkavitch et al., 2018)
Environmental Health	0	None
Family Planning	2	(McKibben et al., 2016; Patil & Janahanlal, 1978)
Genomics	0	None
Global Health	19	(Carrete et al., 2017; De Silva, 2017; Goncalves & Kamdem, 2016; Grove, 2015; Hernandez et al., 2016; Hontelez et al., 2016; Kok et al., 2015; Lee et al., 2016; McGlashan et al., 2016; Meisel et al., 2018; Munar et al., 2015; Nadkarni et al., 2018; Owen et al., 2018; Patil & Janahanlal, 1978; Schuh et al., 2017; Semwanga et al., 2016; Shariatpanahi et al., 2017; Tebbens & Thompson, 2018; Zou et al., 2018)
Health Communication and Health Information Technology	5	(Gillen et al., 2014; Minyard et al., 2014; Ozawa et al., 2016; Siegel et al., 2011; Thomas & Reilly, 2015)
Health Related Quality of Life and Wellbeing	12	(Ahmad & Billimek, 2007; Ahmad, 2005a, 2005b; Ahmad, 2005a, 2005b; Batchelder & Lounsbury, 2016; Brennan et al., 2015; Hoehner et al., 2015; Keane et al., 2015; Lich et al., 2017; Page et al., 2017; Rosas, 2017; Tengs et al., 2001; Yourkavitch et al., 2018)
Hearing and Other Sensory or Communication Disorders	1	(Sheldrick et al., 2016)
Heart Disease and Stroke	1	(Tebbens & Thompson, 2018)
HIV	17	(Anderson & Anderson, 1998; Batchelder & Lounsbury, 2016; Batchelder et al., 2015; Goncalves & Kamdem, 2016; Heidenberger & Flessa, 1993; Hontelez et al., 2016; Kok et al., 2015; Lounsbury et al., 2015; Martin et al., 2015a, 2015b, 2015c; Martin et al., 2015a, 2015b, 2015c; Martin et al., 2015a, 2015b, 2015c; Pedamallu et al., 2012; Rauner, 2002; Weeks et al., 2013, 2017; Yourkavitch et al., 2018; Zou et al., 2018)
Immunization and Infectious Disease	9	(Goncalves & Kamdem, 2016; Hontelez et al., 2016; Osgood, Dyck, et al., 2011; Osgood, Mahamoud, et al., 2011; Ozawa et al., 2016; Rwashana et al., 2009; Schuh et al., 2017; Weeks et al., 2017; Yourkavitch et al., 2018; Zou et al., 2018)
Injury and Violence Prevention	6	(Batchelder & Lounsbury, 2016; Batchelder et al., 2015; Bridgewater et al., 2011; Hovmand & Ford, 2009; Hovmand et al., 2009; Page et al., 2017)
LGBT Health	1	(Kok et al., 2015)
Maternal, Infant, and Child Health	26	(Anderson & Anderson, 1998, Townshend & Turner, 2000, Finegood et al., 2010, Osgood, Dyck, et al., 2011; Osgood, Mahamoud, et al., 2011, Pieters et al., 2011, Ghaffarzadegan et al., 2013, Huang et al., 2013, Wu et al., 2013, Demir et al., 2014, Kumar & Kumar, 2014, Rwashana, Nakubulwa et al. 2014, Sabounchi et al., 2014, Brennan et al., 2015, Edelstein et al., 2015, Frerichs et al., 2015, Hoehner et al., 2015, Keane et al., 2015, Munar et al., 2015, Goncalves & Kamdem, 2016, Ozawa et al., 2016, Semwanga et al., 2016, Schuh et al., 2017, Nadkarni et al., 2018, Pieters, van Oorschot et al. 2018, Tebbens & Thompson, 2018, Yourkavitch et al., 2018)
Mental and Mental Disorders	9	(Batchelder et al., 2015; Bernard et al., 1977; Hosseinichimeh et al., 2018; Kommer, 2002; Lee et al., 2016; Lich et al., 2017; Lyon et al., 2016; Page et al., 2017; Sheldrick et al., 2016)
Nutrition and Weight Status	25	(Finegood et al., 2010, Frerichs et al., 2013, Patrick et al., 2013, Abidin, Mamat et al. 2014, Fallah-Fini et al., 2014, Lan, Chen et al. 2014, Minyard et al., 2014, Sabounchi et al., 2014, Brennan et al., 2015, Frerichs et al., 2015, Hoehner et al., 2015, Keane et al., 2015, Moreland, 2015, Nelson et al., 2015, Thomas & Reilly, 2015, Liu et al., 2016, Soler et al., 2016, Carrete et al., 2017, Jalali et al., 2017, Powell et al., 2017, Rosas, 2017, Frerichs, Lich, et al., 2018; Frerichs, Young, et al., 2018, Frerichs, Lich, et al., 2018; Frerichs, Young, et al., 2018, Meisel et al., 2018, Owen et al., 2018)
Oral Health	2	(Edelstein et al., 2015; Hirsch et al., 2012)
Physical Activity	18	(Davison, Vanderwater et al. 2012; Brennan et al., 2015; Hoehner et al., 2015; Keane et al., 2015; Moreland, 2015; Nelson et al., 2015; Thomas & Reilly, 2015; Liu et al., 2016; McGlashan et al., 2016; Soler et al., 2016; Carrete et al., 2017; Jalali et al., 2017; Powell et al., 2017; Rosas, 2017; Frerichs, Lich, et al., 2018; Frerichs, Young, et al., 2018; Frerichs, Lich, et al., 2018; Frerichs, Young, et al., 2018; Meisel et al., 2018; Owen et al., 2018)
Public health infrastructure	10	(Wu et al., 2013, Crettenden, McCarty et al. 2014, Demir et al., 2014, Kumar & Kumar, 2014, Kok et al., 2015, Ozawa et al., 2016, De Silva, 2017, Schuh et al., 2017, Nadkarni et al., 2018, Pieters, van Oorschot et al. 2018)
Respiratory diseases	2	(Gillen et al., 2014; Osgood, Dyck, et al., 2011; Osgood, Mahamoud, et al., 2011)
Sexually transmitted diseases	21	(Anderson & Anderson, 1998; Batchelder & Lounsbury, 2016; Batchelder et al., 2015; Evenden et al., 2006; Goncalves & Kamdem, 2016; Grove, 2015; Heidenberger & Flessa, 1993; Hontelez et al., 2016; Kok et al., 2015; Lounsbury et al., 2015; Martin et al., 2015a, 2015b, 2015c; Martin et al., 2015a, 2015b, 2015c; Martin et al., 2015a, 2015b, 2015c; Pedamallu et al., 2012; Rauner, 2002; Townshend & Turner, 2000; Viana et al., 2014; Weeks et al., 2013, 2017; Yourkavitch et al., 2018; Zou et al., 2018)
Social determinants of health	21	(Batchelder & Lounsbury, 2016; Batchelder et al., 2015; BeLue et al., 2012; Brennan et al., 2015; Bridgewater et al., 2011; Carrete et al., 2017; Frerichs et al., 2015; Frerichs, Lich, et al., 2018; Frerichs, Lich, et al., 2018; Frerichs, Young, et al., 2018; Frerichs, Young, et al., 2018; Hoehner et al., 2015; Keane et al., 2015; Liu et al., 2016; McGlashan et al., 2016; Moreland, 2015; Nelson et al., 2015; Osgood, Dyck, et al., 2011; Osgood, Mahamoud, et al., 2011; Rosas, 2017; Semwanga et al., 2016; Staller, 2004; Thomas & Reilly, 2015; Yourkavitch et al., 2018)
Substance abuse	8	(Ahmad & Billimek, 2007; Ahmad, 2005a, 2005b; Ahmad, 2005a, 2005b; Batchelder & Lounsbury, 2016; Holder & Blose, 1987; Moxnes & Jensen, 2009; Tengs et al., 2001; Zou et al., 2018)
Tobacco use	5	(Ahmad & Billimek, 2007; Ahmad, 2005a, 2005b; Ahmad, 2005a, 2005b; Soler et al., 2016; Tengs et al., 2001)

We found that fewer works focused on the UN’s SDGs (Table 4). This seems unusual, given that we found 49 of the works focused on global problems and 5 focused on both global and domestic issues. The preponderance of works that did study a SDG focused on the goal to ‘end disease epidemics’ and/or ‘end preventable deaths’. The disease epidemics most commonly addressed were related to STIs.

**Table 4:** United Nations Sustainable Development Goals (UN SDGs)

UN SDG	# of Articles	Article citations
End hunger (Goal #2.1)	0	None
End malnutrition (Goal #2.2)	0	None
Reduce maternal mortality (Goal #3.1)	6	(Pieters et al., 2011; Munar et al., 2015; Hernandez et al., 2016; Semwanga et al., 2016; Nadkarni et al., 2018, Pieters, van Oorschot et al. 2018)
End preventable deaths (Goal #3.2)	26	(Anderson & Anderson, 1998, Rwashana et al., 2009, Wu et al., 2013, Kumar & Kumar, 2014, Rwashana, Nakubulwa et al. 2014, Munar et al., 2015, Batchelder & Lounsbury, 2016, Goncalves & Kamdem, 2016, Hontelez et al., 2016, Ozawa et al., 2016, Semwanga et al., 2016, Soler et al., 2016, Carrete et al., 2017, Page et al., 2017, Schuh et al., 2017, Shariatpanahi et al., 2017, Weeks et al., 2017, Frerichs, Lich, et al., 2018; Frerichs, Young, et al., 2018, Frerichs, Lich, et al., 2018; Frerichs, Young, et al., 2018, Meisel et al., 2018, Nadkarni et al., 2018, Owen et al., 2018, Tebbens & Thompson, 2018, Yourkavitch et al., 2018, Zou et al., 2018)
End disease epidemics (Goal #3.3)	26	(Anderson & Anderson, 1998; Batchelder & Lounsbury, 2016; Batchelder et al., 2015; Evenden et al., 2006; Goncalves & Kamdem, 2016; Grove, 2015; Heidenberger & Flessa, 1993; Hontelez et al., 2016; Kok et al., 2015; Lounsbury et al., 2015; Martin et al., 2015a, 2015b, 2015c; Martin et al., 2015a, 2015b, 2015c; Martin et al., 2015a, 2015b, 2015c; Osgood, Dyck, et al., 2011; Osgood, Mahamoud, et al., 2011; Ozawa et al., 2016; Rauner, 2002; Rwashana et al., 2009; Schuh et al., 2017; Tebbens & Thompson, 2018; Townshend & Turner, 2000; Viana et al., 2014; Weeks et al., 2013, 2017; Yourkavitch et al., 2018; Zou et al., 2018)
Reduce substance abuse (Goal #3.5)	8	(Ahmad & Billimek, 2007; Ahmad, 2005a, 2005b; Ahmad, 2005a, 2005b; Batchelder & Lounsbury, 2016; Holder & Blose, 1987; Moxnes & Jensen, 2009; Tengs et al., 2001; Zou et al., 2018)
Access to sexual/reproductive healthcare (Goal #3.7)	15	(Townshend & Turner, 2000, Evenden et al., 2006, Pieters et al., 2011, Ishikawa, Ohba et al. 2013, Crettenden, McCarty et al. 2014, Viana et al., 2014, Goncalves & Kamdem, 2016, Hernandez et al., 2016, Hontelez et al., 2016, McKibben et al., 2016, Semwanga et al., 2016, Nadkarni et al., 2018, Pieters, van Oorschot et al. 2018, Yourkavitch et al., 2018, Zou et al., 2018)
Eliminate violence against women (Goal #5.2)	3	(Batchelder & Lounsbury, 2016; Hovmand & Ford, 2009; Hovmand et al., 2009)
Eliminate harmful practices against women (Goal #5.3)	1	(Batchelder & Lounsbury, 2016)
Access to sexual reproductive health and rights (Goal #5.6)	1	(Weeks et al., 2013)
Ensure access to adequate and accessible hygiene (Goal #6.2)	0	None
End human trafficking and child labor (Goal #8.7)	0	None
End abuse, exploitation, trafficking, and all forms of violence against and torture of children (Goal #16.2)	1	(Bridgewater et al., 2011)

The majority of works were rated as “high” utility (n = 43) or “low” utility (n = 44) for MCH policy/practice, while 14 were rated as “medium” utility for the field. (Table 1).

### Stakeholder Engagement

In addition to topics, methods and purposes, we also noted patterns in the selected studies regarding stakeholder engagement. While the majority of studies we found (n = 53) did not involve stakeholders in the modeling processes, there were 40 studies which included what we considered to be a high level of stakeholder engagement. A prime example of one of these studies was by Bridgewater et al. (2011), which studied youth violence in Boston and engaged stakeholders throughout the model building and analysis. Qualitative causal loop diagrams developed by the community were used as the basis for a tested/analyzed model with a 12-year time horizon to explore a number of interventions to reduce youth violence.

**Fig. 4:**
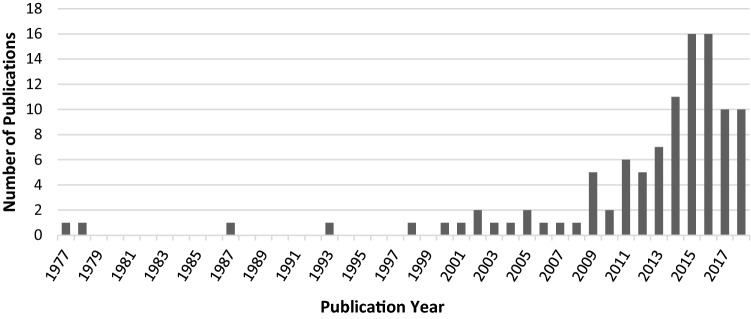
Number of MCH/SD articles published by year

Finally, we found that the number of SD publications on MCH topics has been increasing rapidly in the past decade (see Fig. 4). These works have been spread across 69 publication sources, with the most common being PLOS ONE (n = 7) and Journal of Public Health Management & Practice (n = 7).

## Conclusions for Practice

The application of SD to MCH topics described here include a broad range of approaches, purposes, topics, and levels of stakeholder engagement. The inventory of articles identified in this review provides guidance and direction to those in the MCH workforce looking to bring systems perspectives to their MCH work; however, many areas and approaches remain unexplored.

Qualitative diagramming studies appear to be underused in MCH/SD research. We see opportunities for future studies to draw on qualitative diagrams to bridge science and practice in support of addressing pressing, persistent MCH problems. Group modeling sessions could be integrated into qualitative studies involving in-depth interviews, focus groups, or ethnographic methods (Bridgewater et al., 2011; Hovmand, 2014). Bridgewater et al. (2011) illustrate how stakeholder-engaged group qualitative diagramming can produce insights about the system on its own. This type of qualitative diagramming can also be a stepping-stone for later modeling work. Weeks et al. (2013) illustrate that ethnographic research on MCH topics could be adapted into qualitative diagrams in order to extend their usefulness as drivers of policy. For MCH researchers wary of the mathematical skills necessary for tested/analyzed models, qualitative diagramming can supply a deeper understanding of complex problems in MCH without the time and skill investment of quantitative modeling.

Another future research direction is simulated life course studies, as typified by Osgood, Dyck, and Grassman’s 2011 study of the intra- and intergenerational impact of gestational diabetes on risk of type-2 diabetes. By using historical data to calibrate and validate the model, theories about the intergenerational cycle of risk can be tested. While such studies can never replace longitudinal cohort studies for testing life course theories, they may be able to rule out intergenerational effects if models containing them cannot replicate historical data using the range of parameters estimated in previous studies.

STI research appears to be at the forefront of MCH in terms of adopting SD approaches, possibly because of the similarities between stock-and-flow models and more traditional infectious disease/compartmental models from epidemiology. Childhood obesity has also been a fruitful area for research crossover between MCH and SD; in this case, researchers may have been more comfortable using SD models because they are more common in the biomedical sciences. Collectively, these two fields of research (STI and childhood obesity) have only contributed thirty-five publications, which means many questions regarding the health and wellbeing of MCH populations remain unstudied. One opportunity for MCH researchers and practitioners to lead the way is to incorporate a greater variety of social determinants of health in SD models.

Studies comparing interventions and policies were common, likely because the ubiquity of other modeling methods in comparative cost-effectiveness research makes transitioning to SD methods more acceptable. Unfortunately, many of these studies did not meet basic guidelines – as outlined by the International Society for Pharmacoeconomics and Outcomes Research’s Consolidated Health Economic Evaluation Reporting Standards—for economic evaluation and comparative cost-effectiveness research in terms of reporting style or validation/sensitivity analyses (2000; Sculpher et al., 2000; Garrison, 2003; Weinstein et al., 2003; Husereau et al., 2013). Given the workforce’s role to prioritize actions that make best use of limited resources, assessing the business case for competing interventions is a valuable application of SD methods in the MCH field. However, future research should draw on existing standards for cost-effectiveness research in order to clearly report higher-quality results and best support decisions on resource-allocation.

Finally, MCH professionals should take advantage of teaching and collaboration opportunities inherent in model building. Several studies in this review created interactive, non-intimidating dashboards for their models that laypeople could use with relatively little training (Minyard et al., 2014; Moxnes & Jensen, 2009; Rauner, 2002; Siegel et al., 2011). For some of these projects, the goal was to allow policymakers and public health leaders to try out a number of policy scenarios and receive graphical or simplified feedback on how these policy decisions might affect key outcomes of interest: costs over time, people cured or reached, people missed or harmed, and unintended consequences. In other projects, the goal was to help patients learn how to manage their own health. Interactive models are a way for policymakers, public health leaders, and stakeholders to experiment using methods that deliver consequence-free and evidence-based results in minutes.

The papers in this review demonstrate the potential for the MCH workforce to use SD to understand complex problems such as STI control, obesity, oral health, substance use disorders, and workforce planning. However, many of the wicked problems facing MCH populations, including equity practices, remain unstudied using SD. Furthermore, few of the SD applications described here were then translated into significant action to address the problem under study. These tools have untapped potential. In this critical period of health transformation, SD can produce a better understanding of the varied, multilevel forces interacting to produce the complex problems facing MCH professionals and policymakers.

## Supplementary Information

Below is the link to the electronic supplementary material.

Supplementary file1 (DOCX 22 kb)

## Data Availability

All articles included in this review are accessible online, and the search terms used to query these articles can be found in the Appendix.

## Code availability

The search terms used to query these articles can be found in the Appendix.
