# Supplementary material for: Tools for Supporting the MCH Workforce in Addressing Complex Challenges: A Scoping Review of System Dynamics Modeling in Maternal and Child Health
Source: Matern Child Health J. 2022 Feb 21;26(Suppl 1):176–203. doi: 10.1007/s10995-022-03376-8 (PMC9482604; doi:10.1007/s10995-022-03376-8)
Supplement: Supplementary file 1 — Supplementary file1 (DOCX 22 kb) [file 10995_2022_3376_MOESM1_ESM.docx]

**Appendix 1: Detailed Search Strategies**

System Dynamics sources:

*System Dynamics Review*

Conferences were identified through a conference title search for “system dynamics” NOT vehicle, and selected manually from within the resulting list of conferences. Selected conference entries were:

*Conference on Systems Science Management Science and System Dynamics OR*

*Conference of System Dynamics And Management Science OR*

*International Conference of the Cardiovascular System Dynamics Society OR*

*Western Multiconf of the Soc For Computer Simulation International System Dynamics 1989 OR*

*3rd European System Dynamics Workshop OR*

*Workshop on Applications of System Dynamics In Marketing OR*

*1st European System Dynamics Workshop Or*

*2nd European System Dynamics Workshop OR*

*4th European System Dynamics Workshop OR*

*Euromech Colloquium on Advaces in Simulation of Multibody System Dynamics OR*

*1st International European Forum on Innovation And System Dynamics in Food Networks OR*

*25th International Conference of the System Dynamics Society OR*

*1994 Conference of the International System Dynamics Society OR*

*1994 International System Dynamics Conference OR*

*1995 International System Dynamics Conference OR*

*12th International Conference of the Cardiovascular System Dynamics Society OR*

*Annual Meeting of the System Dynamics Society OR*

*Conference of the System Dynamics Society OR*

*Euromech Colloquium on Advances in Simulation of Multibody System Dynamics OR*

*International System Dynamics Conference OR*

*International System Dynamics Conference 1992*

System Dynamics key word queries:

TS = "system dynamics" AND TS = "model*"

TS = ("model*") AND TS = (("stock*" NEAR/1 "flow*") OR ("stock$?flow$")) OR

TS = ("simulat*") AND TS = (("stock*" NEAR/1 "flow*") OR ("stock$?flow$")) OR

TS = "Causal Loop diagram*"

Health key word queries:

TS = ("Social Work*" OR "Therap*" OR "Disease*" OR "Illness" OR "sickness" OR "Patient" OR "Pharma*" OR "Dental*" OR "Dentist*" OR "Nurs*" OR "Psych*" OR "Physician*" OR "epidemi*" OR "medic*" OR "healthy" OR "doctor*" OR "orthodon*" OR ("oral*" AND "health") OR "obesity*" OR "disabilit*" OR "disabled" OR "clinic*" OR "hospital*" OR "surgery*" OR ("tobacco*" AND smok*) OR (("women*" OR "men*" OR "child*" OR "human" OR "adolescent*") NEAR/1 "health") OR "healthcare" OR "health care" OR "public health" OR "immunization*" OR "polio*" OR "measles*" OR "vaccin*" OR "human health" OR "population health" OR "health concept*" OR "health risk*" OR "national health" OR "health expenditure*" OR (("health" OR "biomedical") NEAR/5 ("polic*" OR "Treatment*" OR "Diagnos*" OR "intervent*" OR "prevent*" OR "outcome*" OR "disabilit*" OR "expenditure*" OR "equit*" OR "disparit*")))

*Indexes=SCI-EXPANDED, SSCI, A&HCI, CPCI-S, CPCI-SSH, BKCI-S, BKCI-SSH Timespan=All years*

TS = ("domestic*" OR "child*" OR "sex*" OR "partner" OR "adolescent*") AND TS = ("abus*" OR "care" OR "violen*")

*Indexes=SCI-EXPANDED, SSCI, A&HCI, CPCI-S, CPCI-SSH, BKCI-S, BKCI-SSH Timespan=All years*

TS = "drug" and TS = ("policy" OR "abus*" OR "prevent*" OR "treat*" OR "intervent*" OR "clinic*")

*Indexes=SCI-EXPANDED, SSCI, A&HCI, CPCI-S, CPCI-SSH, BKCI-S, BKCI-SSH Timespan=All years*

(TS = ("age" or "aging" OR "elder*") AND TS =("policy" OR "policies" OR "care" OR "abus*"))

*Indexes=SCI-EXPANDED, SSCI, A&HCI, CPCI-S, CPCI-SSH, BKCI-S, BKCI-SSH Timespan=All years*

*Search Step 1:* Step one used three different search strategies in the Web of Science Core Citation Indexes (WOS) and PubMed to identify research using SD. SD was defined as causal loop diagramming, stock and flow modeling, system dynamics modeling, or group model building. We searched PubMed as the primary health-related research database and WOS because early searches identified a number of relevant SD applications published on health topics outside of traditional health sciences and health services journals well-indexed in WOS. We searched from 1958-2018 in WOS. The first search strategy was a keyword search of the databases (only articles, books, reviews, proceedings papers, book chapters, notes, and reprints) (see Appendix 1 for detailed search terms). The second search strategy added all works that have been published in SD sources: the journal *System Dynamics Review* and a short list of SD conferences (see Appendix 1 for list of sources). The third strategy added all works that cited four key SD “seed” publications: Forrester (1961-1969), Homer (2006), Sterman (2000-2010), and Sterman (2006). We excluded works that referred to “system dynamics” generally (i.e., “cardiovascular system dynamics”, “family system dynamics”), or that used types of mathematical modeling other than “system dynamics”, “causal loop diagrams”, “stock and flow models”, or “group model building” (i.e., Markov or Susceptible-Infectious-Removed (SIR) models).

*Search Step 2:* Once we identified all of the works in these databases that met the SD criteria, we filtered out those works that did not have a health sciences or health services focus using the following definition: Research on human health outcomes, health behaviors, health services, health workforce, health care and health materials delivery, and health care settings; or research that explicitly discusses a public/population health implication. If a work was published in a health-related source, we assumed it had a health focus. For works not published in health-related sources, we appended health-related key words to our three SD searches (see above for detailed search terms). These key words were nominated by a diverse panel of health practitioners and researchers interested in SD. We excluded works on the internal functions of the human body, works on animals, and works that did not explicitly discuss a health context.

*Search Step 3:* We then reviewed the titles and abstracts (if available) of all works meeting the SD and health criteria. An initial title review by an expert in SD was performed to remove works that clearly had no connection to the field. Two coders then reviewed each abstract to check the first two criteria (SD and health); they also checked that the work was an “application”. We defined an application as any work that describes an SD model thoroughly enough that it could be used as a starting point for model development by another group. We excluded works that simply reviewed the previous uses of SD, described how SD could be used in the future, or described a plan for an as-yet-undeveloped model. The two coders were able to indicate their degree of certainty for their coding decisions; where they were uncertain or disagreed, either a second pair of coders were brought in to arbitrate or the initial pair met to resolve differences.

*Search Step 4:* Identified works meeting the three previous criteria were further reviewed by our research team for their “MCH relevance,” defined as research pertaining to one of the key MCH populations, or one of the key MCH topic areas (see Appendix 2 for detailed definition). Pairs of authors first reviewed abstracts and then full text to determine MCH relevance, with all differences resolved through group discussion. Works that were not deemed relevant to MCH populations or topics were excluded, leaving the final set of works that we found to be MCH-relevant health sciences or health services applications of SD methods.

*Abstraction*: Pairs of authors abstracted information on content and methods from each included work and resolved differences through discussion.

**Appendix 2: Definition of MCH Related**

Articles relating to MCH must meet one of the following criteria:

1. POPULATION: The article must particularly study MCH related populations, which are:

- Pregnant women
- Parents (of children under age 26)
- Infants
- Children under age 18

2. TOPIC: The article must particularly study MCH related topics, which include:

- Disease and health-related outcomes relating to reproduction or pregnancy *(for example: sterility, gestational diabetes)*
- Diseases and health-related outcomes relating to the period surrounding pregnancy and childbirth *(for example: postpartum depression, breastfeeding)*
- Reproductive health broadly, including:
  - Sexually transmitted diseases
  - Contraception
  - Abortion
  - Miscarriage
  - Women’s health and wellness (preconception)
  - LGBT issues
  - Sexual health
  - Sexual development
  - Sex education
  - Sexual violence, including domestic violence and sexual assault
- Provision of services relating to one of the above topics or for one of the MCH populations *(for example: the supply of specialists for children and youth with special health care needs, the distribution of abortion clinics)*

**Rationale Behind the Definition**

The “population or topic” definition allows us to avoid creating a list of conditions to include that will never be exhaustive and that, in the end, we are only interested in when they are studied in infants, children, parents, or pregnant women.

For example, why aren’t accidents included? The answer is that we are only interested in including articles studying accidents if they particularly study them in children.

The list of additional topics is only meant to cover outcomes/diseases/conditions that apply to non-MCH populations.

For example, reproductive health is often studied in non-pregnant non-parents. We are still interested in including these studies despite the fact that they are not conducted in an MCH population.
